# The impact of urine storage methods on the results of quantitative bacterial culture in dog and cat

**DOI:** 10.17221/111/2022-VETMED

**Published:** 2023-03-30

**Authors:** Ching-Jung Lien, Shang-Lin Wang

**Affiliations:** ^1^Graduate Institute of Veterinary Clinical Sciences, School of Veterinary Medicine, National Taiwan University, Taipei, Taiwan, ROC; ^2^National Taiwan University Veterinary Hospital, National Taiwan University, Taipei, Taiwan, ROC

**Keywords:** canine, feline, urinalysis, urinary tract infections

## Abstract

Quantitative bacterial culture (QBC) is the gold standard for determining urinary tract infections. However, the majority of urine samples were sent to a lab for further bacterial culture. Therefore, the storage condition was vital to maintain the quality and accuracy of the urine samples. The main objective of our study was to examine the urine QBC outcomes of (1) immediate culture, (2) culture after urine sample storage at ambient temperature for 24 h, and (3) culture after urine sample storage at 4 °C for 24 hours. There were 49 feline samples and 30 canine samples included in this study. All QBC samples kept at ambient temperature and refrigerator were consistent with immediate QBC in cats. Eight positive results from immediate QBC were in accordance with refrigerated results in dogs. There were ten positive results in the room-temperature sample with two false-positive results. Our study showed that storing conditions at room temperature or refrigeration for 24 h does not impact the results of QBC in cat urine samples. For dog samples, chilled samples have a higher accuracy rate than room temperature samples, although the overall agreement was still satisfactory.

Urinary tract infection (UTI) is a common disease in small animal practice. It has been reported that the incidence of a dog having bacterial UTI throughout its lifetime is about 14%, and female dogs are more often affected than male dogs (Ling 1984). As for cats, bacterial UTI is one of the causes of feline lower urinary tract disease (Kaul et al. 2020). The diagnosis of bacterial UTI is based on the presence of related clinical signs and the detecting pathogen through quantitative bacterial culture (QBC). QBC detects the pathogen and is especially important in diagnosing UTI in cats since bacterial cystitis in the species is uncommon (Weese et al. 2019). Cystocentesis is considered to be the gold standard sampling method for QBC. Immediate inoculation of a urine sample to culture media is recommended for accurate results (Acierno et al. 2018; Weese et al. 2019). However, immediate inoculation may often be infeasible for private practitioners in clinical practice. Therefore, most clinics collected urine samples and store them until transport to a laboratory.

When the urine samples are stored at room temperature, delayed inoculation may lead to false-positive results in 50% of the samples (Padilla et al. 1981). Another study found variations in the total number of bacteria in urine samples after being stored at room temperature for 24 hours (Neumann et al. 2020). On the contrary, some studies revealed a decreased sensitivity of bacterial recovery and an increased false-negative rate with delayed inoculation despite refrigeration (Padilla et al. 1981; Acierno et al. 2018). The first objective of our study was to compare the result of urine QBC between (1) immediate streak plate, (2) streak plate after urine sample stored at room temperature for 24 h, and (3) streak plate after urine sample stored at 4 °C for 24 hours. The second objective was to find out correlations between positive bacterial culture and the result of urinalysis.

## MATERIAL AND METHODS

**Figure 1:**
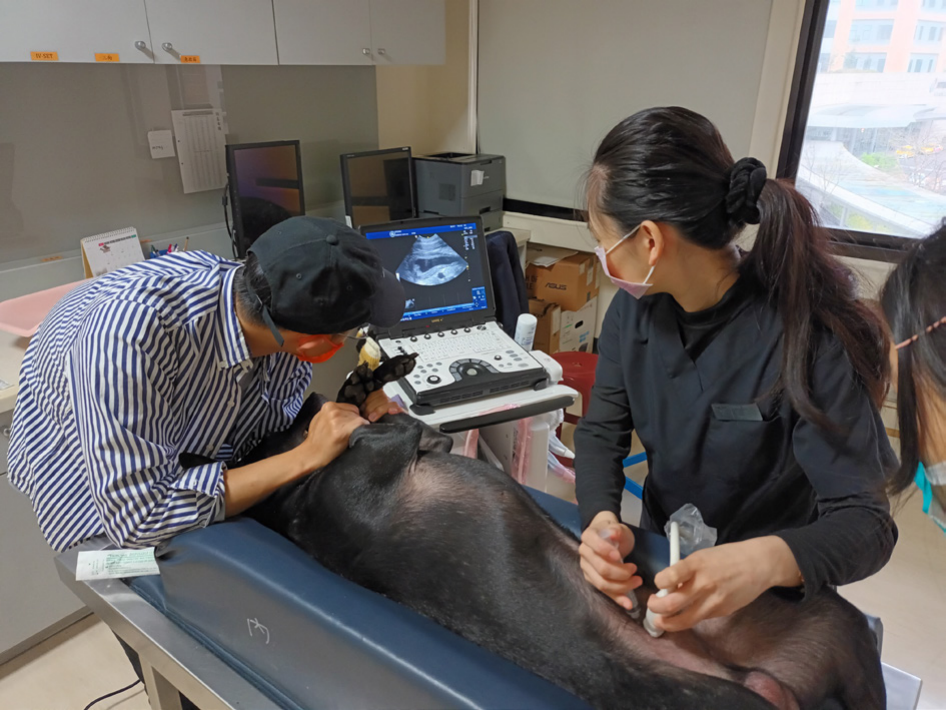
Urine sample collection

This was a prospective study undertaken at our hospital from January 2021 to March 2022. The study was approved by the Institutional Animal Care and Use Committee of the National Taiwan University (Approval No.: NTU-109-EL-00181). Any urine samples collected by cystocentesis from cats and dogs for a urinalysis and QBC were included in this study, no matter the presence or absence of co-morbidities of the patients or the purpose of the hospital visit (Figure 1). Urine samples were expelled into three sterile plastic Eppendorf tubes with 0.5 ml each. One Eppendorf tube was used for QBC immediately. The other two Eppendorf tubes were stored in an air condition-controlled room temperature (24–26 °C) or in the refrigerator (4 °C) for 24 h before QBC. The remaining urine from each sample was used for urinalysis. Each urine sample would be interpreted as an independent event, despite repeated collection from the same patient at several revisits.

The process of bacterial culture is performed in a fume hood and the procedure is briefly described below: a single-use 10 μl inoculation loop was dipped into vortexed urine in the Eppendorf, and then inoculate urine onto a 5% sheep blood agar and a MacConkey agar using streak plate technique. The two agar plates would be incubated at 37 °C for 24 hours. The urine samples stored at room temperature and in the refrigerator would be inoculated after storing for 24 h and using the same process described above.

Positive QBC would be interpreted as more than 1 000 colony-forming units/ml (CFU/ml). Negative QBC would be interpreted as bacterial growth of fewer than 1 000 CFU/ml (Sorensen et al. 2016). For those positive samples, bacterial identification would then be performed using Phoenix^TM^ M50 Automated Microbiology System (BD Diagnostic Systems, Oxford, UK). If there were colonies of different appearances grown on the agar, the different colonies would be re-streaked onto another blood agar and MacConkey agar and incubated for 24 h for bacterial isolation and identification.

A urine sediment wet mount would be examined by microscopy for identifying red blood cells (RBCs), white blood cells (WBCs), and microorganisms. Haematuria is defined as more than 5 RBCs seen under a high-power field (HPF). Pyuria is defined as more than 5 WBCs seen under a HPF. Bacteriuria is defined when bacteria is seen under a HPF (Strachan et al. 2022).

Statistical analyses were performed using a commercial software package (IBM SPSS Statistics v21; IBM, USA). The kappa coefficient was calculated to evaluate the agreement of urine bacterial culture results between immediately-cultured and delayed-cultured (storage at room temperature and refrigerated) samples. For evaluating the relationship between urinalysis and bacterial culture, the results were also calculated as the kappa value. The positive predictive value (PPV) and negative predictive value (NPV) of urinalysis to QBC results were further calculated.

## RESULTS

Seventy-nine urine samples were included in this study. Forty-nine urine samples were collected from cats and 30 samples from dogs. The 49 cat urine samples were collected from 29 individual cats, with 12 females (1 intact, 11 spayed) and 17 males (4 intact, 13 neutered). The 30 dog urine samples were collected from 27 individual dogs, with 11 females (11 spayed) and 16 males (8 intact, 8 neutered).

There were 9 (9/49, 18%) positive results from immediately cultured samples in cats. These 9 positive samples were all infected by single bacteria. The identified bacterial isolates were *Escherichia coli* (7/9, 78%) and *Enterococcus faecium* (2/9, 22%). The culture results and identified bacteria of urine samples that were stored at room temperature and in the refrigerator were totally matched with the immediately cultured results (Table 1).

**Table 1:** Urine quantitative bacterial culture results in 49 cats

	Methods		
Culture result	Immediate culture	Culture after stored at 4 °C for 24 h	Culture after stored at room temperature for 24 h
Positive	9	9	9
Negative	40	40	40

There were 8 (8/30, 27%) positive results from immediately cultured samples in dogs. These 8 positive samples were all infected by single bacteria. The identified bacterial isolates were *E.* *coli* (4/8, 50%), *Proteus mirabilis* (3/8, 37.5%), and *Pseudomonas aeruginosa* (1/8, 12.5%). There were 10 (10/30, 33%) positive results from the samples stored at room temperature for 24 hours. These 10 positive results were all infected by single bacteria. The identified bacterial isolates were *E.* *coli* (6/10, 60%), *Proteus mirabilis* (3/10, 30%), and *Pseudomonas aeruginosa* (1/10, 10%). Eight positive samples from the immediately cultured group yielded the same results after being stored at room temperature for 24 hours. Two (2/22, 9%) negative samples from the immediately cultured group yielded positive culture results with both single *E.* *coli* infections. However, the agreement of culture results between immediate culture and culture after stored at room temperature for 24 h was excellent (kappa 0.842). The culture results and identified bacteria of urine samples stored in the refrigerator were totally matched with the immediately cultured results (Table 2).

**Table 2:** Urine quantitative bacterial culture results in 30 dogs

	Methods		
Culture result	Immediate culture	Culture after stored at 4 °C for 24 h	Culture after stored at room temperature for 24 h
Positive	8	8	10
Negative	22	22	20

Haematuria (RBC > 5 cells/HFP) was present in 8 (8/49, 16%) cat samples, there was no relationship between positive QBC result and the presence of haematuria (kappa –0.067). Pyuria (WBC > 5 cells/HPF) was present in 9 (9/49, 18%) samples, and the agreement between positive QBC result and the presence of pyuria was high (kappa 0.864). The PPV and NPV of pyuria for QBC were 89% and 98%. Bacteriuria was present in 8 (8/49, 16%) samples, and the agreement between positive QBC result and the presence of bacteriuria was almost perfect (kappa 0.929). The PPV and NPV of bacteriuria for QBC were 100% and 98%.

Haematuria was present in 9 (9/30, 30%) dog samples, and the relationship between positive QBC result and the presence of haematuria was weak (kappa 0.098). Pyuria was present in 13 (13/30, 43%) urine samples, and the agreement between positive QBC result and the presence of pyuria was moderate (kappa 0.502). The PPV and NPV of pyuria for QBC were 54% and 94%. Bacteriuria was present in 8 (8/30, 27%) samples, and the agreement between positive QBC result and the presence of bacteriuria was high (kappa 0.83). The PPV and NPV of bacteriuria for QBC were 88% and 95%.

## DISCUSSION

Current guidelines for delayed processed urine QBC indicated that, if the sample is unable to be cultured immediately, it should be refrigerated and cultured within 24 hours (Weese et al. 2019). This suggestion is based on some studies comparing immediate and delayed processed QBC results of dog urine samples stored under different temperatures or transport tubes (Padilla et al. 1981; Patterson et al. 2016; Acierno et al. 2018). However, in the aforementioned study, the urine samples were not collected from natural live patients (Patterson et al. 2016). It may not contain inflammatory mediators and cells to reflect true conditions and might affect bacterial survival and growth. Furthermore, only one bacterial isolate, *E.* *coli*, and dog urine samples were evaluated in that study which may not provide a broad conclusion in the clinical practice. Other studies evaluated the impact of different storing conditions on urine QBC results. However, these studies had several limitations. Most studies collected dog urine samples only and some studies mixed dog and cat data for calculation (Padilla et al. 1981; Acierno et al. 2018; Coffey et al. 2020). Some studies included voided urine samples for QBC, which was discouraged since contamination was possible (Coffey et al. 2020; Neumann et al. 2020; Hedstrom et al. 2021). Some of the studies used preservatives such as boric acid which was not often used in clinical practice (Rowlands et al. 2011; Hedstrom et al. 2021).

We simulated actual conditions of clinical practice in this study. All urine samples for QBC were only collected by cystocentesis and the urinalysis was performed right after urine collection. The urine samples for QBC were inoculated after storing them for 24 h, simulating the time needed to send urine samples to external laboratories.

We calculated dog and cat data individually to see whether there was a different result in different species since cat and dog urine possess different properties that may influence the ability to against urinary pathogens.

Our study indicated that there was little difference in delayed processed QBC results from different storage conditions. The QBC results of cat urine samples storing under different temperatures for 24 h were all identical with immediately cultured samples. The QBC result of dog urine samples storing at 4 °C for 24 h was identical with immediately cultured samples, and the kappa agreement between immediately cultured urine samples and urine samples storing at room temperature for 24 h was still excellent. However, storing urine samples at room temperature leads to a few more false-positive results.

The reason for the different results of dogs and cats can be assumed to be the different urine properties of different species. One study discovered microbiota in the urine of healthy dogs, but there were no published studies that found similar results in healthy cats (Burton et al. 2017). It is reasonable to believe that urine microbiota is different in different species, thus the aetiology of bacterial UTI and interaction between host defence and pathologic bacteria may also be different.

“Active urine sediment” is defined as urine sediment with the presence of haematuria, pyuria, or bacteriuria, and is thought to be closely related to positive QBC results (Strachan et al. 2022). Our study result indicated that urine sediment with the presence of bacteriuria was highly correlated with positive QBC results in both dogs and cats; pyuria was moderately and highly correlated with positive QBC results in dogs and cats, respectively. This result was similar to the previous studies (Bailiff et al. 2008; Martinez-Ruzafa et al. 2012). However, we found that haematuria was less associated with positive QBC results.

As for pyuria, it had both high PPV (89%) and NPV (98%) for cat QBC. However, dog QBC had a high NPV (94%) but a lower PPV (54%). Pyuria indicates active inflammation but is not always associated with infection (Callens and Bartges 2015). Some diseases such as chronic kidney disease, feline idiopathic cystitis, and urolithiasis result in urinary tract inflammation, which is characterized as haematuria, pyuria, and proteinuria, but without evidence of an aetiologic agent. On the other hand, patients with certain co-morbidities, such as cancer patients receiving chemotherapy, may have bacteriuria without concurrent inflammation. A previous study evaluated correlations between urinalysis and positive QBC results and revealed that pyuria had only 64% PPV in cats, but had high NPV in both dogs and cats (Torre et al. 2022). In our study, we did not record the patients’ concurrent disease, so the underlying cause of low PPV cannot be evaluated. However, we can speculate that, according to high NPV, if the patient is not immunocompromised, the absence of pyuria is highly correlated to a negative QBC result.

In our study, we found haematuria weakly associated with positive QBC results in both dogs and cats. The possible reason for this finding is that haematuria can be present in many situations other than UTI, such as urolithiasis, trauma or neoplasia of the urinary tract, feline idiopathic cystitis, prostatic diseases, sterile haemorrhagic cystitis. A recent study also found that haematuria has both lower PPV and specificity for predicting a positive QBC (Torre et al. 2022).

Another possible reason was that most of the urine samples for urinalysis in our study were collected by cystocentesis, blood contamination was possible during the collecting process, thus affecting the result of haematuria.

The correlation between QBC results and clinical signs of UTI was also evaluated in this study. Significant differences were founded in the clinical signs of UTI and QBC results in cats and dogs (*P* < 0.001 and *P* = 0.013). The sensitivity and specificity of clinical signs of UTI were 100% and 77.5% in cats, and 100% and 50% in dogs. The low specificity in both species majorly resulted from the diseases of diabetes mellitus, chronic renal failure, feline idiopathic cystitis, and sterile haemorrhagic cystitis after cyclophosphamide administration. Therefore, bacterial culture should always be performed in patients with UTI signs before antibiotic treatment.

There were several limitations in our study. Firstly, there were 49 and 30 urine samples from cats and dogs, respectively. However, only 9 (9/49, 18%) samples from cats and 8 (8/30, 27%) samples from dogs were positive for QBC, and those were all infected by single bacterial isolates. Therefore, we couldn’t investigate the impact of the interaction between multiple bacterial isolates on delayed processed QBC. The results of no statistically significant difference between each delayed culture group were also possibly due to small numbers of positive QBC results. Second, positive cat QBC urine samples were majorly (7/9, 78%) infected by *E.* *coli*, the impact of different bacterial isolates and different biological conditions might not be shown in our study. Third, the condition of refrigeration and the room temperature was set at 4 °C and 24–26 °C, respectively. However, in reality, “room temperature” varies depending on different seasons. Furthermore, the temperature in transport trucks may be much higher than the temperature in the free air, especially in the summer. These may all impact the results. Lastly, we did not count the actual CFU of each positive QBC result. One study showed significant differences in CFU from different storage conditions. However, it did not impact the interpretation results of “positive” or “negative” (Acierno et al. 2018).

In conclusion, our study revealed that storing conditions at room temperature or refrigeration for 24 h does not impact the results of QBC for cat urine samples. As for dog samples, those stored in refrigerated condition has more accuracy rate than those stored at room temperature, but the overall agreement was still satisfactory. The presence of pyuria and bacteriuria were highly correlated with positive QBC results and the absence of pyuria and bacteriuria could rule out the possibility of positive QBC results in cats. The absence of pyuria correlated with negative QBC results in dogs. Haematuria was less valuable to predict QBC both in cats and dogs.
